# On the Virtues of Colchicum in Chorea

**Published:** 1816-09

**Authors:** T. Raven

**Affiliations:** House Pupil to the Norfolk and Norwich Hospital.


*For the London Medical and Physical Journal.*

### *On the Virtues of Colchicum in Chorea;* by T. Raven, House Pupil to the Norfolk and Norwich Hospital.

THOUGH the power of Tinct. Colchici to give ease in the gout is well known, yet, never having heard of its being found a remedy for Chorea Sancti Viti, I have taken the liberty of sending you three cases, in which it has proved most wonderfully successful, without increasing any of the secretions, or teazing the patients any more than the same quantity of pure water would have done.

On May 4, 1816.—Samuel Steward, æt. 8, was admitted into the Norfolk and Norwich Hospital, under Dr. Alderson, on account of Chorea Sancti Viti, with which he had been most violently afflicted for more than three months. The morning after his admission, he was ordered to take the following powder:—

℞ Pulveris Jalapii, ℈j.
Cambogiæ et
Hydrargyri submuriatis ana. gr. iij. m. fit pulvis.

And, after the operation of this medicine, Tinct. Colchici Autumn. (secundum Want) m. x. 6tis. horis. He had not taken it quite a week before he was cured, and would have been discharged, had not the doctor desired he might remain some time longer in the house, lest the disease should return; as it did not, he took his discharge on Saturday, June 18th, 1816.

On Saturday, May 24, 1816—Sarah Day, æt. 13, but who had never menstruated, was admitted under the same physician, for for the same very formidable disease as the boy's: how long she had laboured under it, neither she nor the person who came with her could say; she was made to pass through the same course of medicines as the boy, only that she took m. xx. 6tis. horis. She too, like him, was restored to health in three or four days, and was kept in the house about the same time as he was.

On Saturday, June 15, 1816, Ann Burner, æt. 12, was admitted into the same house, under Dr. Alderson, afflicted with the same terrible disease; and the doctor, encouraged by the good success of the two former cases, prescribed the same drops, viz. m. xx. 6tis horis. It had no visible effect upon any of the secretions, only that it kept her bowels moderately open; a fortnight after, he ordered her to increase the dose to m. xxv. which produced nausea and vomiting, together with three very loose stools: upon decreasing the dose and continuing it three or four days longer, she was restored to her former good state of health, and was discharged cured, July 27, 1816.

If you think these cases may be of service, I have no doubt that you will publish them in your very valuable Journal. Of the good effects of Tinct. Colchici in the Rheumatism, chronic as well as acute, (for the gout we never see in our Hospital,) I may take some other time to give you an account.

*Norwich;*
*July* 27, 1816.

P.S.—The tincture was prepared according to the ingenious Mr. Want's prescription, given in your Journal.

